# Projection the long-term ungauged rainfall using integrated Statistical Downscaling Model and Geographic Information System (SDSM-GIS) model

**DOI:** 10.1016/j.heliyon.2019.e02456

**Published:** 2019-09-17

**Authors:** N.N.A. Tukimat, N.A. Ahmad Syukri, M.A. Malek

**Affiliations:** a Faculty of Civil Engineering and Earth Resources, Universiti Malaysia Pahang, Malaysia; b Earth Resources and Sustainability Center (ERAS), Universiti Malaysia Pahang, Malaysia; c Institute of Sustainable Energy (ISE), Universiti Tenaga Nasional, Malaysia

**Keywords:** Engineering, Environmental science, Earth sciences, GIS, Ungauged rainfall, SDSM, Statistical downscaling, Kriging

## Abstract

An accuracy in the hydrological modelling will be affected when having limited data sources especially at ungauged areas. Due to this matter, it will not receiving any significant attention especially on the potential hydrologic extremes. Thus, the objective was to analyse the accuracy of the long-term projected rainfall at ungauged rainfall station using integrated Statistical Downscaling Model and Geographic Information System (SDSM-GIS) model. The SDSM was used as a climate agent to predict the changes of the climate trend in Δ2030s by gauged and ungauged stations. There were five predictors set have been selected to form the local climate at the region which provided by NCEP (validated) and CanESM2-RCP4.5 (projected). According to the statistical analyses, the SDSM was controlled to produce reliable validated results with lesser %MAE (<23%) and higher R. The projected rainfall was suspected to decrease 14% in Δ2030s. All the RCPs agreed the long term rainfall pattern was consistent to the historical with lower annual rainfall intensity. The RCP8.5 shows the least rainfall changes. These findings then used to compare the accuracy of monthly rainfall at control station (Stn 2). The GIS-Kriging method being as an interpolation agent was successfully to produce similar rainfall trend with the control station. The accuracy was estimated to reach 84%. Comparing between ungauged and gauged stations, the small %MAE in the projected monthly results between gauged and ungauged stations as a proved the integrated SDSM-GIS model can producing a reliable long-term rainfall generation at ungauged station.

## 1 Introduction

Nowadays, the global warming is unavoidable. Uncontrolled emissions dispersed to the atmosphere as a main factor in increasing the greenhouse gasses (GHGs) level and contribute to the climate changes crisis. Nor Aizam and Peter (2011) stated the most river in Malaysia were frequently affected by flood event due to the unpredictable rainfall variability. In the year of 2016, the majority part of east coast of Peninsular Malaysia had been flooded after couple weeks of continuous rain started on Nov, 2014. Most of the areas which near to the river were flooded because of abnormal rainfall intensity which 60% higher than the average monthly rainfall during normal condition. The downstream rivers became overflow caused by heavy rainfall at an upstream areas.

Focused on Kuantan River Basin, an availability of rainfall stations were limited and cater only at small parts of the entire basin. Besides, the quality of the data records was also one of the major concerned and become critical especially during disaster event. Based on the data provided, there were less than 50% of missing rainfall data especially on Nov and Dec (flood events) which can be as a challenging problem among analyst and policy makers. Lacking in the rainfall records will having large impact to the long-term hydrological modelling (Adrián and Rolando, 2018).

A large number of statistical approaches such as linear, nonlinear and hybrid methods have been tested and improvise in effort to generate the rainfall at ungauged station. However, the hydrological characteristics and physiometeorological variables are having complex relationship which could not simply presenting in linear and nonlinear mechanism (Ouali et al., 2017; Xue and Gui, 2015). Zeynoddin et al., 2018 proved the hybrid methods which combining the linear stochastic models with extreme learning machine (ELM) methods has good performance in improving the accuracy of rainfall projection. Meanwhile Piman and Babel (2016) suggested to use transposition and regionalization techniques in Hec-HMS in treating the rainfall-runoff estimation at ungauged station and proved the error between observed and estimated were less than 11%.

Thus, statistical downscaling model (SDSM) has been introduced to understand the changes on present and long-term future climate condition in responding to the long term dispersion of GHGs and aerosol emission into the atmospheric system. It was categorised as hybrid model which implementing the model output statistics and perfect prognosis. The local climate changes trend can be projected only at the region that having particular rainfall station.

In the climate assessment, the general circulation models (GCMs) were implemented as an climatic agent which concerned the potential GHGs in the long term. The GCMs is a mathematical modeling to describe the general circulation of the atmosphere and ocean characteristics. It can be as a platform to monitor the climatic responses in the context of the GHGs. GCMs is cover the topography area up to 50,000 km^2^ radius. The Canadian Climate Data and Scenarios (CCDS) is a climate research center which provides climate models and observational data named Coupled Model Intercomparison Project Phase 5, CMIP5 (AR5). The AR5 was used to generate the local climates concerned with the potential radiation in the long term. The advantage of AR5 is the model does not requires an additional flux adjustment in producing a good simulation. It requires a resolution combination of the atmospheric and oceanic component in producing a good agreement in a prediction. The SDSM application potentially to provide reliable result while having limited sources (Shahid et al., 2017). Its implementations were also well documented and has been successfully tested in numerous studies especially for the long term Malaysia's climate assessment (Noor et al., 2019; Tarmizi et al., 2019; Tahir et al., 2018; Tukimat et al., 2018).

Meanwhile, geographic information system (GIS) can be used to interpolate spatial attribute between available stations in the reasonable scale. In this case study, the attribute refer to the long term climate changes trend at ungauged station. Interpolation is the procedure used to predict cell value for location that lack sample points. There were many geostatistical interpolation methods available such as Thiessen polygon, Inverse Distance Weighting (IDW), linear regression and Kriging. Mair and Fares (2011) proved the Kriging method was successfully to produce the lowest error and better accuracy in the predictions compared to other interpolation methods. Comparison of the Kriging method interpolation map with grided isohyet data indicate that the areas of the greatest rainfall deficit were confined to the mountainous region of west Oahu. One of the advantage in the Kriging is the model weightage is not only based on the distance between the measured points and the prediction location but also on the overall spatial arrangement of the measured points. To use the spatial arrangement in the weights, the spatial autocorrelation must be quantified. Thus, in Kriging, the weight, depends on a fitted model to the measured points, the distance to the prediction location, and the spatial relationships among the measured values around the prediction location (ESRI, 2016).

Therefore, the integrated SDSM-GIS model has been used to examine the accuracy of the long-term projected rainfall at ungauged station. It is very important to evaluate the accuracy of the projected rainfall mapping at ungauged area.

## 2 Study area

**Fig. 1:**
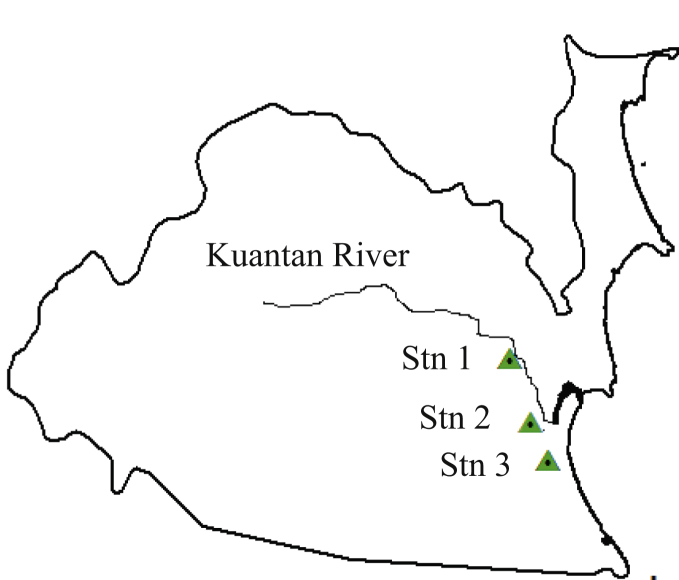
Kuantan river basin.

The study area was focused on Kuantan River Basin. There were 3 rainfall stations which near each other had been selected to achieve the objective of the study. It was also based on the availability of 30 years length data record (1984–2013) which having least missing data. The selected rainfall stations were Pam Paya Pinang station (Stn 1: 3832015), Paya Besar station (Stn 2: 3732020) and Kg Sg Soi station (Stn 3: 3732021) as shown in Fig. 1.

The boundaries cover 102^o^ 23′E and a 3^o^ 53′N. The total area of the state is 35965 km^2^, which is 9.8% of the total area of Peninsular Malaysia. The state of Pahang consists of diverse surface area, ranging from sea levels of 0 m–3000 m above sea level. Approximately 70% of the country comprises of low-density land and those lands are less than 200 m above sea level. About 30% of this land was flooded before. The average wind speed at this area during the flood was 6 mph and had the average humidity of 80%. The rainfall distributions have two monsoons known as North-East and South-West monsoons. The average annual rainfall was in range of 2000 mm/year to 4000 mm/year and the range of total average temperature was 27 °C–32 °C. The average monthly rainfall in this area ranges from 200 mm to 790 mm per month.

## 3 Methodology

**Fig. 2:**
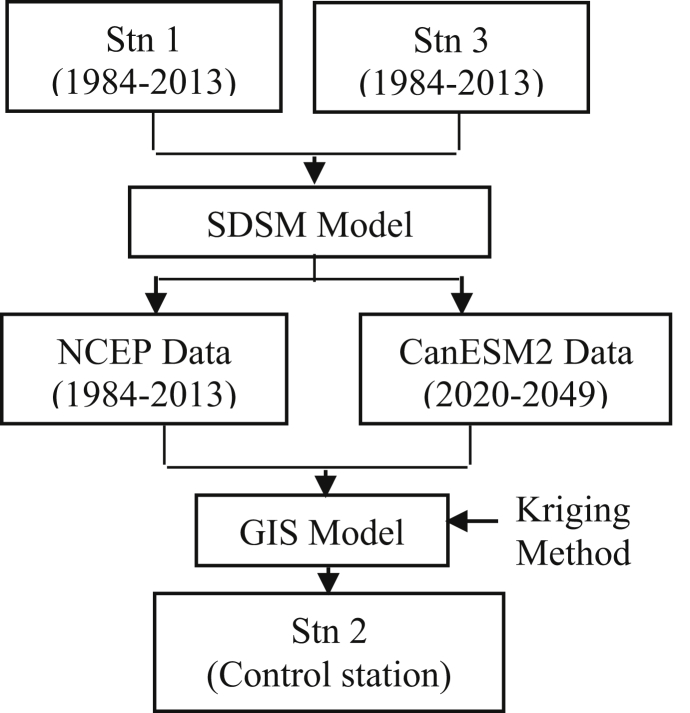
Methodology of the study.

Fig. 2 explains the methodology of the study. There were 2 phases in this analyses; 1) simulation phase and 2) projection phase. In the simulation phase, there were 5 predictors (atmospheric characteristics) which provided by NCEP data required to form the rainfall equation for each rainfall stations. The predictors’ selection were based on the monthly correlation values (R) between predictor-predictand (rainfall) relationships. The performance of the rainfall equation was tested during calibration and validation processes using statistical downscaling model (SDSM).

Next, the Kriging method by GIS was performed to interpolate the climate trend at Stn 2 which located in between of Stn 1 and Stn 3. This process was repeated during second phase (projection phase) for projected climate result. However, this time, the same predictors name which provided by CanESM2 Data have been used to analyse the projected climate results.

### 3.1 Climate projection by SDSM

The SDSM was introduced by Wilby and Dawson (2007) and widely used in the hydrological issues in varies climate scenarios. The model provides station scale climate information from the grid resolution GCM-scale output using multiple regression techniques. Its build up the relationship between GCMs’ variable (so called as predictors) and the local scale variable acts (so called as predictants). The SDSM was categorized as a hybrid model. It utilized a linear regression method and a stochastic weather generator. A 30-years length period or more as standard references recommended for climate change and climate variability study in climatology (Simon et al., 2015).

It consists of two steps: 1) determining of the whether rainfall occurs on each day and 2) determining of the estimated value of rainfall on each rainy day with considered estimated GHGs. Subsequently, the predictor-predictand equations were developed using multi-linear regression approach for generation the long-term climates at the region. The rainfall (y) on day t can be determined by:

(1) $$y_{t}=F^{-1}\left[\varphi Z_{t}\right]$$

(2) $$Z_{t}=\beta_{0}+\sum_{j=1}^{n}\beta_{j}{\hat{u}}_{t}+\beta_{t-1}+\varepsilon$$

Where F is the empirical function of $y_{t}$, ∅ is the normal cumulative distribution function, $Z_{t}$ is the z-score on day t, β is the regression parameter, ${\hat{u}}_{t}$ is normalized predictor and ε is the variable parameter. For the rainfall analysis, the equation was transformed to the fourth root to take account for the skewed nature of the rainfall distribution.

The rainfall and temperature were modelled using stochastic weather generator based on the selected predictors. The large-scale predictors for the meteorological projection employed by the SDSM model were referred to the NCEP reanalysis for calibration and validation processes and CanESM2 for the long-term generation. Refering to the IPCC Fifth Assessment Report (AR5), there were 3 RCPs have been used in this study to provide plausible future scenarios of anthropogenic forcing spanning a range from a low emission scenario characterized by active mitigation (RCP 2.6), through two intermediate scenarios (RCP 4.5 and RCP 6), and to a high emission scenario (RCP 8.5).

Although the statistical downscaling has several limitations (Wangsoh et al., 2017), however the SDSM model does not require high computational demand to view the simulation results but has ability to produce high quality of projection results. These advantages, as a whole, had made SDSM a reliable tool for climate downscaling (Samadi et al., 2013, Tukimat and Harun, 2015) and was selected as a downscaling tool to generate the future climate trend at the study site.

### 3.2 GIS-Kriging interpolation

A GIS was basically a computerized information system like any other database, but with an important difference which mean all the information in GIS should be linked to a geographic spatial references such as latitude and longitude, or other spatial coordinates. According to the Environmental Protection Agency a GIS works by combining database functions with computer mapping to map and analyses geographic data. It uses a layering technique to combine various types of data. Special GIS software was used to analyses layered data and create new layer of data. Geographical was a geographic reference, means it referred to data of spatial coordinates on the surface of the earth map. Information system data base of attribute data corresponding to spatial location and procedure to provide information for decision making.

GIS consists of two components which were spatial component and attribute component. Spatial component defined as the location of an information. Basically it was constructed from three forms which were lines, points, and polygons. Spatial data was categorized into two which were lines, points and polygons. Spatial data was categorized into two which were in raster and vector. Individual cells in a matrix, or grid, format was used in the raster data to represent real world entities. It was obtained from satellite from satellite imagery, aerial images of space, and a map scan. Meanwhile, the coordinate was used in the vector data to store the shape of spatial data object. It was performing in CAD software, Shapefile, Map info table, delimited text file with coordinates, Triangulated Irregular Network (TIN). Attribute component was the information in the database. The information that was mentioned before was related to geographic information, the position and size of plots of land, the systems network of road, and railways, drainage, sewerage, ranked rivers and building.

Interpolation is the procedure used to predict cell value for location that lack sample points. The Kriging method, the weights are based not only on the distance between the measured points and the projection location but also on the overall spatial arrangement of the measured points. To use the spatial arrangement in the weights, the spatial autocorrelation must be quantified. Thus, in Kriging method, the weight, depends on a fitted model to the measured points, the distance to the projection location, and the spatial relationships among the measured values around the projection location (ESRI, 2016). The equation of the Kriging as stated below (Setianto and Triandini, 2013).

(3) $$\bar{Z}\left(so\right)=\sum_{i=1}^{N}\lambda i.Z\left(si\right)$$

Z(si) = the measured value at the location
λi = an unknown weight for the measured value at the location
So = the projection location
N = Number of measured values

Analysis of rainfall data was based on the analysis of space and time. For the analysis of space, location of rainfall stations was plotted by using GPS, then the amount of annual rainfall was plotted by each rainfall station. After that, the isohyet maps that represented the annual rainfall for each year was predicted by using the interpolation of Kriging method in GIS software. This interpolation is the procedure used to predict cell value for location that lack of sample points. The aim of isohyet map development using GIS was to identify the distribution of rainfall patterns and to compare the rainfall distribution between years. In addition, the changing patterns of rainfall from year to year can also be analysed. Other than that, the analysis of changes in rainfall per year was analysed by using the graph changes in rainfall for each station. The analysis was then performed to identify the highest rainfall received during the review period and then the causes and effects of the highest rainfall were identified.

## 4 Results and discussion

### 4.1 Calibrated and validated of climate

There were 5 predictors have been selected based on the stronger monthly R performances; surface zonal velocity (p_u), surface vorticity (p_z), temperature (temp), relative humidity at 500hpa (r500) and geopotential height at 850hpa (p850). The R values were 0.72–0.81 which closer to 1.0. These predictors set were used to form the rainfall equations at each region.

**Fig. 3:**
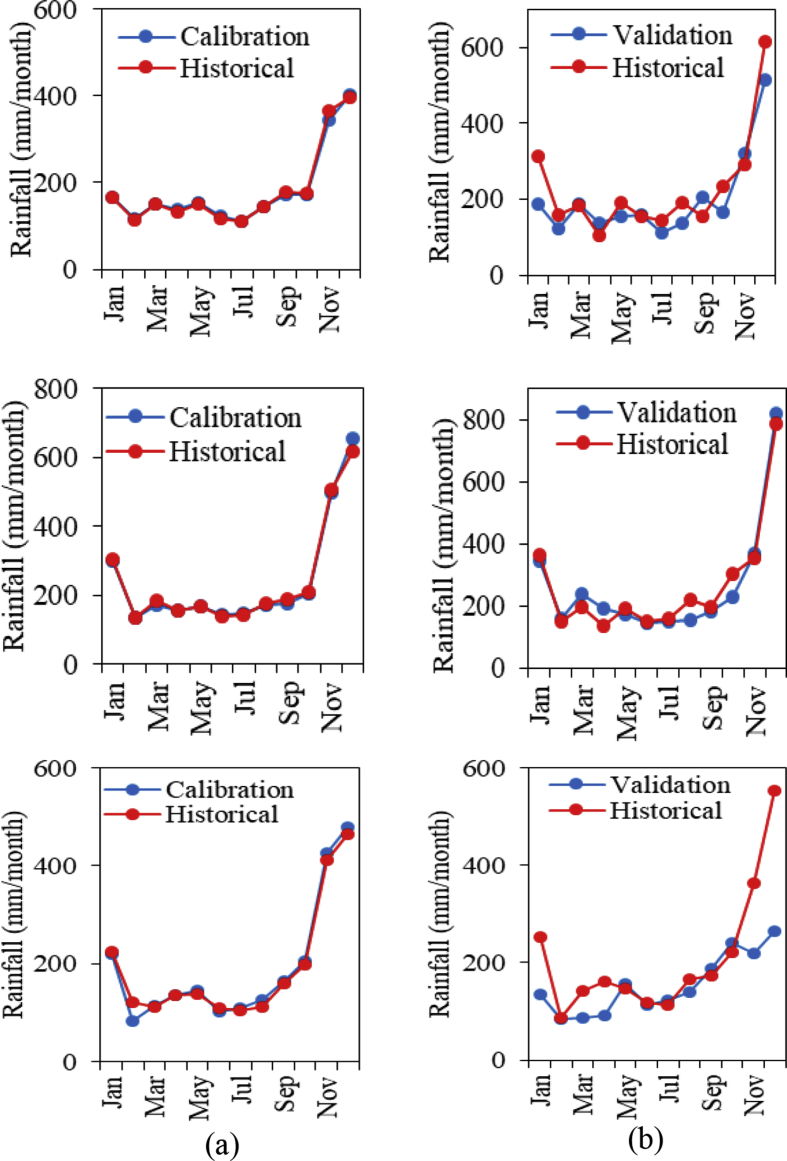
Results of (a) calibration (1984–1998) and (b) validation (1999–2013) performances with the historical data for 3 rainfall stations (top: Station 1, middle: Station 2, bottom: Station 3).

The performances of these equations were evaluated during calibration and validation processes as shown in Fig. 3. According to the results, all stations were successfully to perform well in calibrated and validated results with minimum error. These performances as stated in Table 1. The best simulated result was at Stn 2 because successfully to produce the smallest %MAE with 2.6% and 13.7% in the calibrated and validated results, respectively. Meanwhile at Stn 3, the %MAE in the validated result was estimated greater reaches 23.2%. The St.D shows the closer wide dispersed range between calibrated and validated results except at Stn 3. However, all stations were obtained higher R values as a proved the predictors’ selection were well organised and huge influence with the local climates.

**Table 1:** Statistical analyses for calibrated and validated performances.

Stations	St.D		MAE (%)		R	
	Cal	Val	Cal	Val	Cal	Val
Stn 1	0.5	0.5	2.2	21.3	1.0	0.7
Stn 2	0.8	0.9	2.6	13.7	1.0	0.8
Stn 3	0.6	0.3	6.2	23.2	1.0	0.7

### 4.2 Climate projection by Δ2030s

**Fig. 4:**
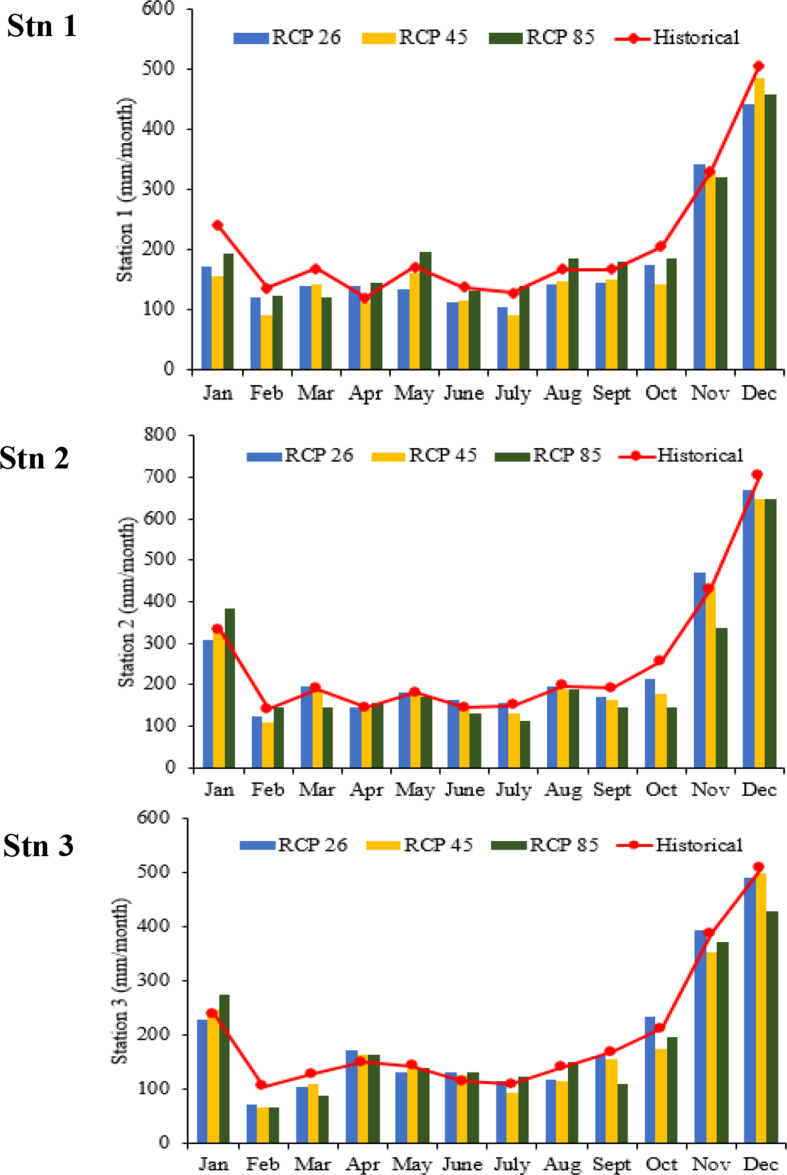
Comparison between projected rainfalls by all RCPs with the historical rainfall trend.

The local climates were projected using same predictors set however this time provided by CanESM2. Based on Fig. 4, all the RCPs agreed that the long term annual rainfall potentially to produce lesser annual rainfall intensity compared to the historical in all stations. The months of Feb to Aug were predicted to have reasonable amounts of rainfall in all scenarios and the least rainfall projection which are scenario RCP4.5 at Stn 1 with decrement -14.11% due to the Southwest monsoon, which normally dominates the dry season period. Meanwhile the projected rainfall result was expected to increase to occur during Northeast monsoon especially in November and December.

Rainfall trend at Stn 2 which projected by RCP2.6 recorded as the highest rainfall intensity compared to other stations and reached to 2995 mm/year. However, this intensity still lower 2.14% compared to the historical record. While the lowest rainfall intensity was occurred at Stn 1 with 2120 mm/year which expected to decrease 14.11% compared to the historical data. Meanwhile for the Stn 2 and Stn 3, RCP 8.5 shows the least rainfall changes in the long term with -11.32% and -6.5%, respectively compared to other RCPs.

### 4.3 Climate interpolation by GIS

**Fig. 5:**
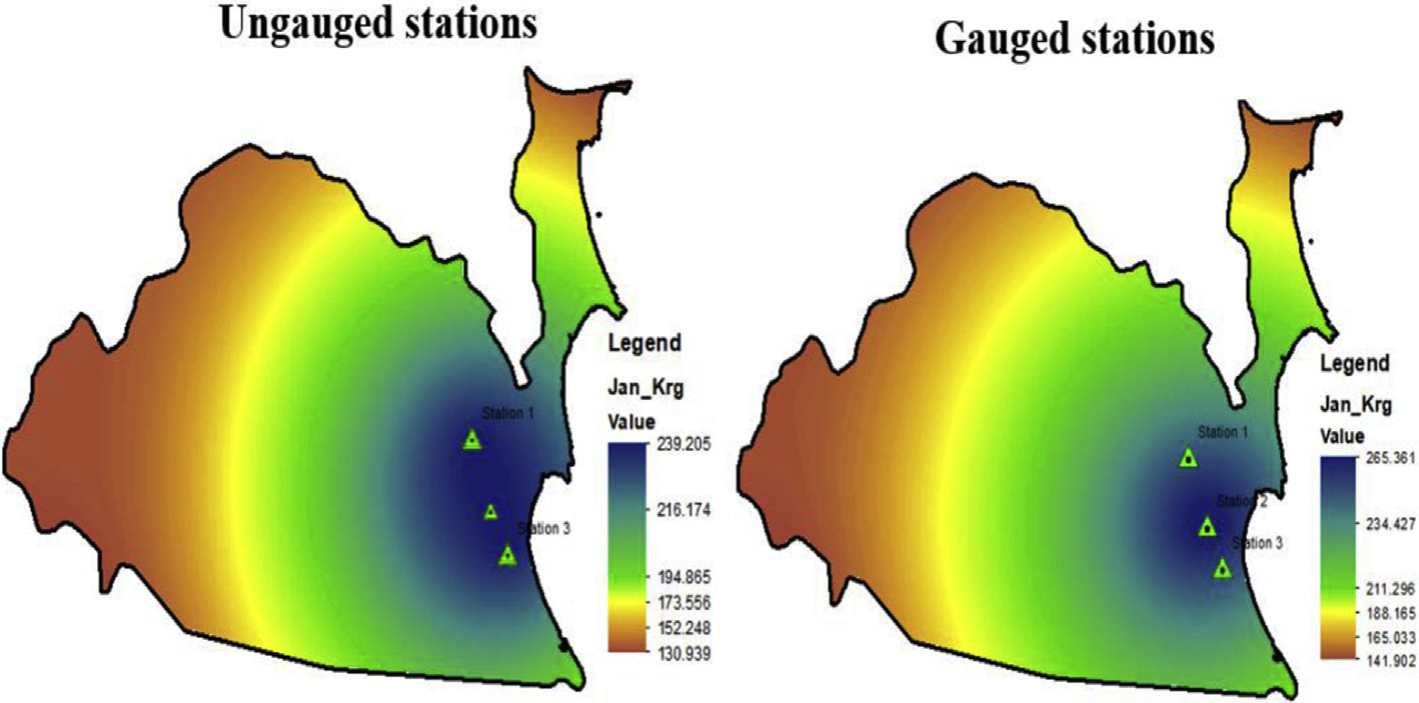
Comparison of historical monthly rainfall intensity on Jan between ungauged and gauged rainfalls trend.

The GIS-Kriging method used to treat the ungauged station. In this case study, the Stn 2 being as a control station which reacted as an ungauged station. Fig. 5 indicates the comparison of the historical rainfall distribution between gauged and ungauged rainfall stations.

From the analyses, the highest %MAE between ungauged and gauged station was occurred in Feb with 16.1%. Even the error was slightly bigger however the rainfall distribution pattern at ungauged station was still consistent to the gauged station. Meanwhile month of April was succesfully to produce the lowest error with 0.1% compared to the other months. The monthly rainfall for this month at ungauged station was in range of 135.43 mm until 148.7 mm compared to the gauged station was in range of 132.86 mm until 148.38 mm. March, May, and June were also produced lower percentage error with less than 5%. Thus, as general the interpolation by GIS-Kriging was succesfully to produce climate trend which consistent to the historical trend.

**Fig. 6:**
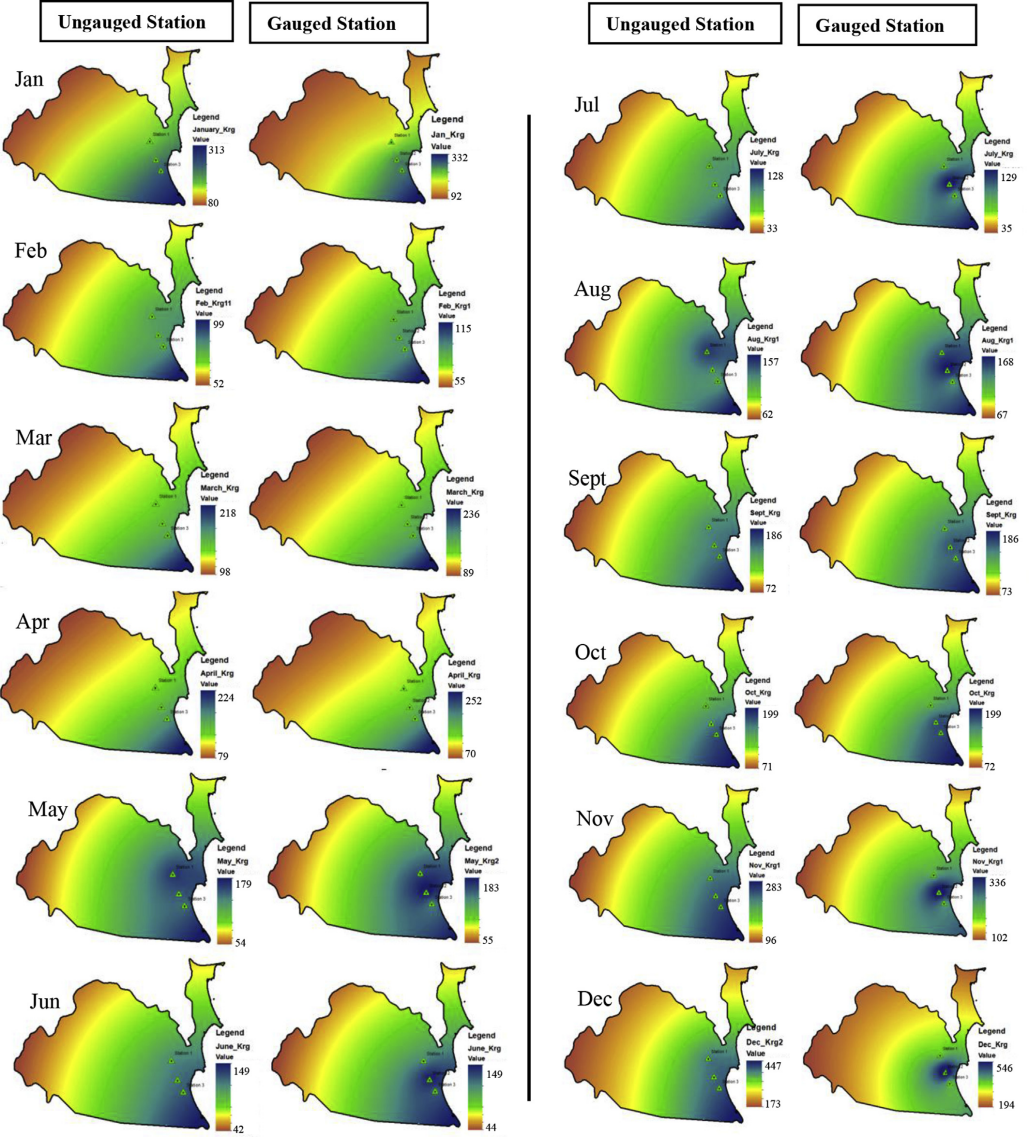
Comparison pattern of monthly rainfall distribution during year Δ2030 between ungauged and gauged stations (unit in mm/month).

Meanwhile Fig. 6 shows the comparison of projected monthly rainfall by RCP 4.5 for the Δ2030s between ungauged and gauged stations. In this study, the RCP4.5 was performed because it produced closer simulated result compared to the rest RCPs. The RCP4.5 is referred to the radiative forcing at 4.5 Wm^-2^. It provides a common platform for climate models to explore the climate system response to stabilizing the anthropogenic components of radiative forcing (Van Vuuren et al., 2011).

The monthly projected rainfall distribution by ungauged station were succesfully to produce very small %MAE, which were <1% for every month. The rainfall patterns were also very closed to rainfall pattern provided by gauged station. The lowest of %MAE was recorded in October (0.1%). The range of rainfall intensity at ungauged station was 167.58mm–199.05mm, while at gauged station, the range of rainfall intensity was in range of 167.33mm and 198.99mm. Consistent trend were occurred in May, June, July and September with <5% of error.

However, the higher errors was estimated to occur on December whereby the %MAE between ungauged station and gauged station was reaches 15.3%, The monthly rainfall for ungauged station in December was within 479.67mm–547.37mm compared to the gauged station which in between of 538.38mm and 646.50mm. Followed by Feb whereby the %MAE reaches to 13.9%. Eventhough, all these critical month were still succesfully to produce consistent rainfall distribution pattern to the gauged station with reasonable errors.

## 5 Conclusion

The finding of this study showed the SDSM-GIS model has huge potential to generate the long term rainfall pattern at ungauged station. The Kriging method can be performed as interpolation agent to treat the ungauged station area.

The SDSM was succesfully to provide the long term climate pattern at the gauged stations with lesser %MAE and higher R value closed to 1.0 in the simulated results. Due to the projected results, the rainfall intensity were estimated to reduce in average 14% in Δ2030s compared to the historical. In general, All the RCPs agreed to produce the similar rainfall patern throughout a year with small intensity changes. However, the increment/decrement was varies in different level of radiation forcing. The RCP2.6 and RCP8.5 were recorded as the highest and least rainfall changes in the long term records.

The GIS was succesfully to treat the ungauged station. In this study, Stn 2 was used as control station which reacted as ungauged station. The interpolation results produced by GIS-Kriging at ungauged station was slightly similar to the control station with %MAE was 16.1% (historical comparison) and 15.3% (projection comparison). It proven the integrated SDSM-GIS model can provides the rainfall trend at ungauged station reaches 84% of accuracy.

## Declarations

### Author contribution statement

N. N. A. Tukimat: Conceived and designed the experiments; Analyzed and interpreted the data; Wrote the paper.

N. A. Ahmad Syukri: Performed the experiments; Analyzed and interpreted the data.

M. A. Malek: Analyzed and interpreted the data; Contributed reagents, materials, analysis tools or data.

### Funding statement

This work was supported by Universiti Malaysia Pahang (grant vot RDU1803156), Malaysian Meteorological Department (MMD), Drainage and Irrigation Department, Malaysia (DID), and iRMC Bold2025, Universiti Tenaga Nasional, Malaysia (grant code: RJO 1043 6494).

### Competing interest statement

The authors declare no conflict of interest.

### Additional information

No additional information is available for this paper.
